# Supplementary material for: Functional Analysis of Long Non-Coding RNAs Reveal Their Novel Roles in Biocontrol of Bacteria-Induced Tomato Resistance to Meloidogyne incognita
Source: Int J Mol Sci. 2020 Jan 30;21(3):911. doi: 10.3390/ijms21030911 (PMC7037896; doi:10.3390/ijms21030911)
Supplement: Supplementary file 1 [file ijms-21-00911-s001.zip › ijms-69008-SI-to conversion/Supplementary Figures.pdf]

Supplementary Figures:

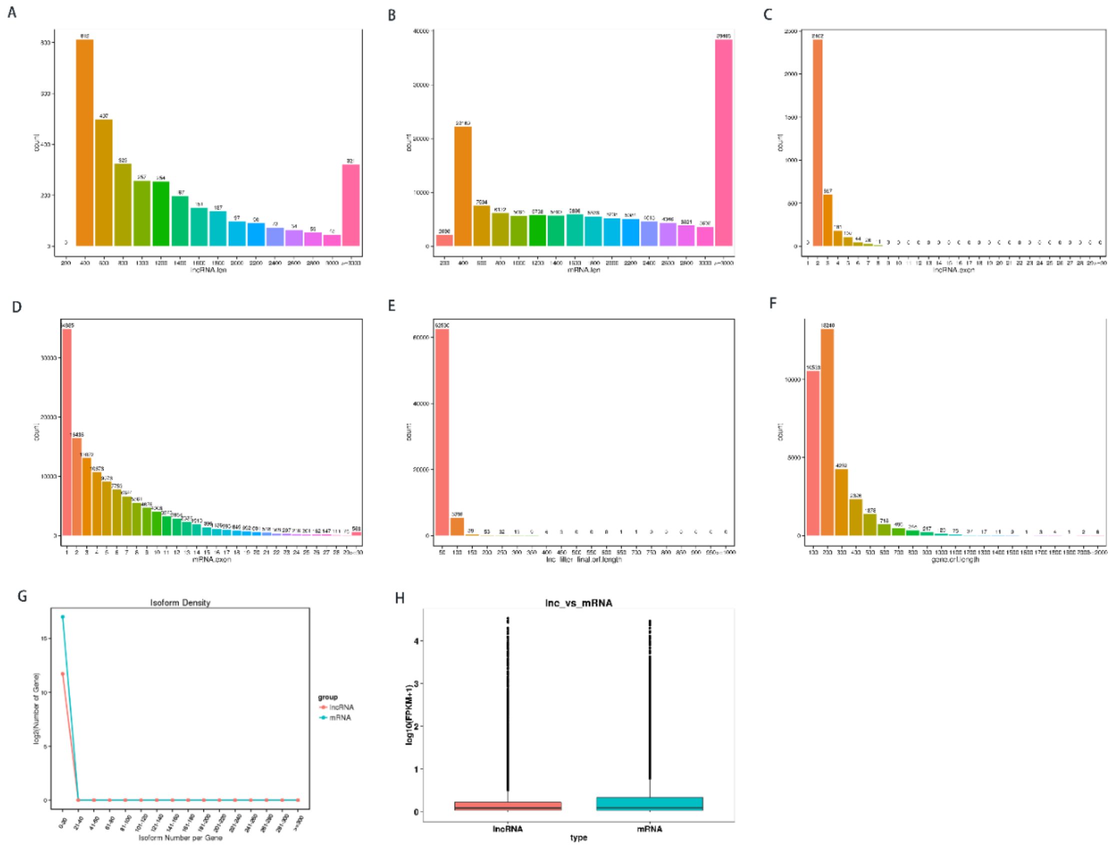

Figure S1. Structural feature and expression analysis of tomato lncRNAs. (**A**) The length of lncRNAs. (**B**) The length of protein coding genes. (**C**) The exon number of lncRNAs. (**D**) The exon number of protein coding genes. (**E**) The ORF length of lncRNAs. (**F**) The ORF length of protein coding genes. (**G**) The alternatively spliced isoforms of lncRNAs and protein coding genes. (**H**) The expression level of lncRNAs and protein coding genes.

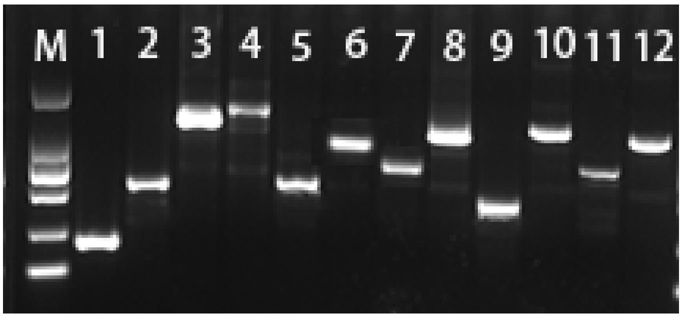

Figure S2. RT-PCR confirmation of tomato lncRNAs. Lane 1-12 means selected lncRNAs. Lane 1 is lncRNA18894, Lane 2 is lncRNA21563, Lane 3 is lncRNA24059, Lane 4 is lncRNA25797, Lane 5 is lncRNA35115, Lane 6 is lncRNA39939, Lane 7 is lncRNA8668, Lane 8 is lncRNA44664, Lane 9 is lncRNA45969, Lane 10 is lncRNA48734, Lane 11 is lncRNA51612 and Lane 12 is lncRNA7183.
